# Future opportunities in solar system plasma science through ESA’s exploration programme

**DOI:** 10.1038/s41526-024-00373-9

**Published:** 2024-03-14

**Authors:** Mats Holmstrom, Mark Lester, Beatriz Sanchez-Cano

**Affiliations:** 1 Department of Physics, Umeå University, SE-901 87 Umeå, Sweden; 2 Swedish Institute of Space Physics, P.O. Box 812, SE-981 28 Kiruna, Sweden; 3 School of Physics and Astronomy, University of Leicester, University Road, Leicester, LE1 7RH UK

**Keywords:** Plasma physics, Atomic and molecular physics

## Abstract

The solar wind interacts with all solar system bodies, inducing different types of dynamics depending on their atmospheric and magnetic environments. We here outline some key open scientific questions related to this interaction, with a focus on the Moon and Mars, that may be addressed by future Mars and Moon missions by the European Space Agency’s Human and Robotic Exploration programme. We describe possible studies of plasma interactions with bodies with and without an atmosphere, using multi-point and remote measurements, and energetic particle observations, as well as recommend some actions to take.

## Introduction

The solar system is embedded within the expanding solar atmosphere, i.e., the solar wind, a tenuous plasma, consisting mostly of protons and electrons, continuously emitted by the Sun. Each body that encounters the solar wind will interact with this plasma; this interaction depends on a number of characteristics of the body, such as the presence and strength of an internal magnetic field, the presence, density, composition of an atmosphere, and the size of the planet and its rotation rate^1^. Some bodies such as the Moon and Mars’ moon Phobos spend part of their orbit in the solar wind and part in the space environments of their respective planets. The solar wind, however, is not a steady flow of plasma as the sun itself is highly variable. This variability can lead to a number of different transients. Coronal mass ejections (CMEs) are dense bubbles with strong magnetic field embedded that are ejected from the Sun. The solar wind also have regions with different velocities - fast streams with speeds up to about 1000 km/s and slow streams that can have velocities down to about 300 km/s - that can interact when they propagate out in the solar system. Solar energetic particle events (SEPs) are bursts of protons and electrons that are accelerated in the Sun’s atmosphere during solar flares (bursts of emitted energy in all of the electromagnetic spectrum), or accelerated by shocks. All of these phenomena interact with planetary environments in different ways. This variability leads to the concept of Space Weather, which is the influence of the plasma interactions of the solar wind on planetary magnetospheres, ionospheres, atmospheres and surfaces, and the subsequent impact on humans and technologies. To study these interactions, experience at Earth demonstrates that it is necessary to have many sensors at different locations and specifically at least one set of observations continuously in the solar wind.

In the Sections that follow, we first describe key gaps in our knowledge of how the solar wind interacts with bodies in the Solar System. Then we discuss priorities for the space programme by outlining observations that could fill these gaps, with recommendations summarized in Table 1.

**Table 1:** Recommendations in the short, middle and long term for addressing some fundamental scientific question within the ESA HRE programme

Open fundamental scientific question	Focus of ESA HRE platform : LEO, Moon, Mars, BLEO	Context of related recent and future space experiments	Short. middle or long term
Evolution of CMEs through heliosphere? Multi-point measurements	Moon, Mars	Magnetometer, Small plasma packages, energetic particles	Short
Plasma surface interactions?	Moon, Mars (Phobos)	Small plasma packages, ENA imagers, energetic particles	Short (Moon), Mid - Long (Phobos)
How are atmospheres lost into space?	Moon, Mars	Small plasma packages, UV and ENA imagers, radio science, energetic particles	Short - Mid

## Key knowledge gaps

### How does plasma interact with the surface at bodies without any significant atmosphere?

In its orbit around the Earth, the Moon is exposed to different plasma regions: The undisturbed solar wind, the shocked solar wind in Earth’s magnetosheath plasma, and the tenuous plasma in the tail region of Earth’s magnetosphere. Due to the lack of atmosphere, the plasma will precipitate directly onto the surface regolith of the Moon. The Moon does not have a global magnetic field like Earth, but has local magnetic fields. At regions with such crustal magnetic anomalies the plasma will interact and be modified by these localized crustal fields. The precipitating plasma will modify the composition of the exposed surface, and will also sputter charged and neutral particles. At the Moon it has been observed that 1% of the solar wind protons are reflected back into space^2,3^, and that 20% are reflected as neutral hydrogen atoms^4^. The precipitating solar wind will also weather the surface and change its optical properties^5^. All details of the microphysics of these plasma-surface interactions are still not known. The small moons Phobos at Mars is in a similar way exposed to different plasma environments in its orbit around the planet, from the undisturbed solar wind, to shocked solar wind, and plasma in the tail of Mars’ induced magnetosphere^6^. Both moons are also exposed to heavy ions from the ionosphere of their parent bodies when they pass through the tails of the magnetosphere’s these ions becoming implanted in the surface and also causing sputtering of particles^7,8^.

### How are atmospheres lost into space?

Where did the water on Mars go? Geological features on the surface of Mars indicate the presence of oceans on its surface in the past. Why are Earth and Mars so different today? Both are in the habitable zone, where liquid water can be sustained, given sufficiently large atmospheric pressure. When the solar system formed, the planets were probably quite similar, but today Mars is cold and dry with a thin atmosphere, compared to Earth^9^. One channel of atmospheric loss is the escape of ions through the interaction between the upper atmosphere and the solar wind^10^.

The solar wind propagates outward from the sun and is mostly composed of protons and electrons, with an embedded magnetic field. When the solar wind meets a planet there will be an interaction, that will depend on the magnetization of the planet. A planet will have an intrinsic magnetic field if it has a core that can sustain a dynamo that generate sufficiently strong electric currents inside the planet. This creates a magnetosphere that can hold off the solar wind. This is the case at Earth. If the intrinsic magnetic field is weak, or non-existent, an induced magnetosphere will be created by electric currents in the ionosphere. This is the case at present for Venus and Mars^11^.

However, Mars probably had an intrinsic magnetic field for some time during the planet’s first billion years, but now only has localized crustal magnetic fields^12^.

At Mars, there is also a significant escape of neutrals through thermal (Jeans) escape^13^ (the hottest atoms reach escape velocity) and by photochemical processes^14^(chemical reactions that give atoms escape velocity). Recent observations suggest that neutral escape is the dominant escape process for present day Mars^14^. This is due to the relatively small mass of Mars, but for extreme solar wind conditions, that were more common for the early Sun^15^, ion escape may have been larger. Understanding atmospheric escape today through observations also allows us to extrapolate back in time to gain knowledge of how the atmospheres have evolved through the history of the Earth and Mars. Such understanding of these processes in our solar system, solidly based on observations, also allows us to predict atmospheric loss at extrasolar planets. The question of atmospheric escape is also related to the question: Do Magnetospheres protect planets? The traditional view is that intrinsic magnetic fields protect atmospheres from erosion by the solar wind^16^.

This is formalized by the concept of magnetic limited escape, a regime of atmospheric loss where the strength of the magnetic field determines the atmospheric loss rate^17^. This view also results in simplistic formulas for the loss rate over time^16^. It is a natural idea, since the magnetic field creates a magnetosphere around which the solar wind is deflected, and therefore cannot directly erode the atmosphere.

**Fig. 1:**
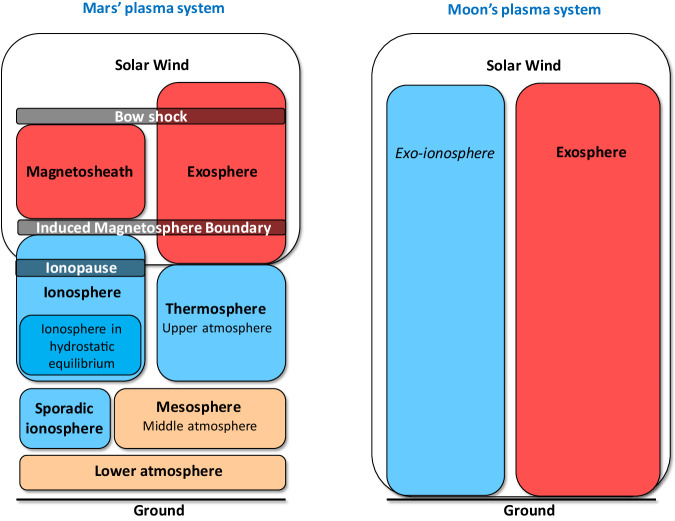
The different regions of the interaction between the solar wind and (left) an un-magnetized planet, like Mars, and (right) an object without an atmosphere, like the Moon. For Mars, the solar wind is thermalized at the bow shock, creating the magnetosheath. Below the induced magnetosphere boundary (IMB) we have planetary ions in the ionosphere, and at the ionopause we have a pressure balance with the solar wind. For neutrals, we have the collisionless exosphere in direct contact with the solar wind. At the Moon we only have a collissionless neutral exosphere, created mostly from sputtering at surface, since the solar wind can precipitate directly onto the surface. Charged particles near the Moon create an exo-ionosphere^45^, and quickly escape to space or precipitate onto the surface, since they are subject to the solar wind electromagnetic fields.

However, the view that magnetospheres protect planets has recently been challenged by observations that ion escape from Venus, Earth and Mars are of similar magnitudes, even though the magnetization of the planets are different^18^. The processes of atmospheric escape are different for induced and intrinsic magnetospheres. The solar wind comes into direct contact with the upper parts of the atmosphere in the case of an induced magnetosphere, allowing escape to space over large regions, since ions at the top of the atmosphere are accelerated by the electromagnetic fields to escape velocities. This is illustrated in Fig. 1, where we show the regions and boundaries from space down to the surface at Mars. For an intrinsic magnetosphere, much of the atmosphere is shielded from interaction with the solar wind. At the cusp regions of the magnetosphere (near the north and south poles at Earth), the solar wind can however precipitate onto the atmosphere, depositing energy. In the cusps it is also possible for ions to be accelerated and escape to space. In principle the total escape to space could be of similar magnitude for an induced magnetosphere as for an intrinsic magnetosphere with a larger flux from a smaller area in the case of the intrinsic magnetosphere, and a smaller flux over a larger area for the induced magnetosphere. This would explain the fact that the observed escape of ions to space from Venus, Earth, and Mars is of similar magnitude (10^25 ^ions/s)^19^. However, we have to remember that these planets do not only differ in magnetization, but also in solar wind conditions and atmospheric mass and composition.

These ideas were first put forward in a conference presentation by Barabash^18^, and were also discussed later that year by Strangeway et al.^20^. It has then been elaborated by Brain et al.^21^, summarized by Ramstad and Barabash^19^, and the implications for the evolution of Earth’s atmosphere^22^ and the evolution of exoplanets^23^ has been discussed. However, limited quantitative analysis of the idea has been done. Some theoretical scaling analysis was done by Blackman and Tarduno^24^, and later by Gunell et al.^25^.

Knowledge of the role of intrinsic magnetic fields for atmospheric escape is also important for studies of the evolution of exoplanet atmospheres. At present we mostly have to draw conclusions from studies in the Solar System, that we can extrapolate to exoplanets. Observations of ion and neutral escape at Mars can be done from orbit around the planet. In orbit around the Moon, or even on the surface, escaping particles from the Earth can continuously be observed when the Moon is inside Earth’s magnetosphere.

### Multi-point measurements in plasma systems

Making measurements at different points in the plasma environment provides information on the temporal effect and response as well as spatial variability of plasma systems and processes which we are, otherwise, unable to determine from single point measurements and statistical studies. Earth and Mars are the only planets in our solar system where multi-point measurements have to-date been possible for any extended period. At both planets this has now left us with a host of unanswered questions, in particular regarding the solar wind interaction with the planetary plasma during CMEs and high speed/slow speed streams. At Mars for example, in addition to the question of atmospheric loss (see above), we also have limited understanding of many physical processes relating to the coupling between the neutral and ionized gases. For example, how does plasma penetrate the nightside regions of the Martian system, where there is no source of ionization^26,27^. Transport from the dayside is one possible mechanism, but it is unclear what processes drive that transport and whether this is steady or variable. Another possible mechanism is electron particle precipitation guided by the twisted nature of the Martian tail which is a consequence of crustal magnetic field interaction with the solar wind^28,29^. The patchy nature of the plasma regions may suggest that there is a natural variability but whether this is driven or how it is driven by the solar wind is unclear. On the dayside, spatial structures in the plasma occurs in a number of areas, but it is clear that the ionosphere can often have large depletions of plasma density above a certain altitude^30^. Enhanced solar wind pressure and interplanetary magnetic field direction, in particular, appears to be important for the formations of structures and depletions in the ionosphere, but on what timescale, and over what spatial scales this occurs on the dayside is unclear^30,31^.

Our knowledge of the evolution of structures propagating through the inner heliosphere is incomplete^32^. This is mainly due to a lack of multi-point measurements at different distances from the sun as well as at different angular separations. Different radial distances provide information on the speed as well as evolution of the structure of CMEs as they move through the heliosphere. Different angular separations provide information on the angular extent in the heliosphere, which can then be compared directly with the measurements of the active region at the sun. Combining the two gives a 2-D analysis of the evolution in the ecliptic plane, which currently is only possible using models.

Space is also a great laboratory for plasma physics, due to the low densities and thereby few collisions. The Lunar wake is a good example, where plasma expansion into vacuum can be studied^33^. Fundamental and applied questions can also be investigated by active experiments that release or accelerate particles^34^. This can be studied both near the Moon and Mars. Surface impactors on bodies without a significant atmosphere, e.g., the Moon, is another example of modifying the environment for scientific studies.

### Radiation and energetic particle environment at the Moon and Mars

The radiation environment at the Moon and Mars requires continuous observation, from orbit and from the surface, if we want future human presence in these environments, as well as understanding the response of their plasma environments as secondary particles (generated from collisions from external energetic particles) may further interact with the ambient environment (i.e., atmosphere at Mars, and the surface regolith at Mars and the Moon). There are a number of energetic particle sources that need to be understood in order to determine the temporal variability of a particle radiation environment^35^. For example, there is a well-defined long-term solar cycle variation of the Galactic Cosmic Rays (GCR) at both locations, as well as at Earth, such that the dose rate is a minimum at the peak of the solar cycle, and a maximum at the lowest point of the solar cycle. This long-term variation is altered during CME events, which “shield” the planet from GCRs and tend to occur more often and more intense during the maximum of solar activity^35^. In addition, a major source of sporadic and potentially high intense particle precipitation are Solar Energetic Particles (SEP) events, which also are more likely to occur during solar maximum when the GCR flux is lower. Studies of the CMEs themselves are also important because they are sources of hazardous energetic particles, even more harmful in short term than the galactic rays^36^. Not only the flux of energetic particles should be investigated, but also the spectral characteristics, since it also governs health effects on humans. Variability is related to transient events propagating through the solar wind, such as Coronal Mass Ejections (CME), where the magnetic field becomes enhanced. The propagation of such events is of direct interest for all planetary bodies and moons which interact with the solar wind, as well as the understanding of how such events evolve as they propagate further into the heliosphere^37^.

## Priorities for the space programme

### Multi-point measurements

To undertake the necessary investigations of the solar wind interactions, we require solar wind measurements at the same time that measurements are being made close to Mars or the Moon (when the Moon is inside Earth’s magnetosphere). Key measurements that should always be taken are solar wind velocity and density, from which the solar wind plasma pressure can be calculated, and the IMF (Interplanetary Magnetic Field), magnitude and direction. In addition, a UV instrument to measure ionizing photons would be beneficial. These instruments could be placed on orbiters which have apogees outside the bow shock of the Martian system (which guarantees enough daily solar wind coverage), or near the moon Phobos, which has an orbit of 8 h and is often in the solar wind. To investigate the evolution and structure of CMEs, we would envisage a small package of key instruments to include a magnetometer, plasma package (electrons and ions from suprathermal to very energetic levels) and a radiation monitor/energetic particle detector, such as the engineering instruments flown on missions such as Rosetta, BepiColombo and JUICE^38,39^, but also more dedicated science instrumentation to characterize the high energy plasma regime.

### Remote observations

Multi-point observations cannot give the full global picture of the plasma environment. We also require remote imaging of the plasma and neutral species. This can be for example UV imaging of extended hydrogen exospheres^40^. Another example is the imaging of energetic neutral atoms (ENAs) that allows remote monitoring of plasma, since the neutrals are produced by charge exchange between the plasma and neutrals^41^. At objects without a significant atmosphere, e.g., the Moon, surface emissions of ENAs can also be remotely monitored^4^. Remote observation of ionospheres can also be accomplished by radio science experiments^42,43^. Ionospheric height profiles can be deduced from the refraction of radio waves. An example would be a transmitter on Phobos, communicating with satellites in Mars orbit, or with Earth.

## Outlook and summary

We have outlined opportunities for future observations in solar system plasma science through the European Space Agency’s Human and Robotic Exploration (HRE) programme, summarized in Table 1 as recommendations in the short, middle and long term, at the Moon and at Mars.

The proposed experiments would operate on the surface and in orbit of the Moon and Mars. Such observations would advance the science targets listed in Table 1, and would enable the ESA’s HRE programme to make significant contributions in answering these open fundamental scientific question in solar system plasma science.

## Data Availability

Data sharing is not applicable to this article as no datasets were generated or analysed during the current study.
